# Supplementary material for: KLF5 loss sensitizes cells to ATR inhibition and is synthetic lethal with ARID1A deficiency
Source: Nat Commun. 2025 Jan 8;16:480. doi: 10.1038/s41467-024-55637-5 (PMC11711288; doi:10.1038/s41467-024-55637-5)
Supplement: Supplementary file 1 — Supplementary Information [file 41467_2024_55637_MOESM1_ESM.pdf]

SUPPLEMENTARY INFORMATION

KLF5 loss sensitizes cells to ATR inhibition and is synthetic lethal with *ARID1A* deficiency

Samah W. Awwad[1,2#], Colm Doyle[1], Josie Coulthard[1], Simon Lam[1,2], Nadia Gueorguieva[1,2], Aldo S. Bader[1,2], Vipul Gupta[2], Rimma Belotserkovskaya[1,2], Tuan-Anh Tran[1], Shankar Balasubramanian[1,3], and Stephen P. Jackson[1,2#].

[1]*Cancer Research UK Cambridge Institute, University of Cambridge, Cambridge, UK*
[2]*The Gurdon Institute and Department of Biochemistry, University of Cambridge, Cambridge, UK*
[3]*Yusuf Hamied Department of Chemistry, University of Cambridge, Cambridge, UK*
[#] Correspondence: samah.diab@cruk.cam.ac.uk, steve.jackson@cruk.cam.ac.uk.

- **Supplementary Figures**
- **Supplementary Tables**
- **Supplementary Methods**
- **Supplementary References**

Supplementary Figures:

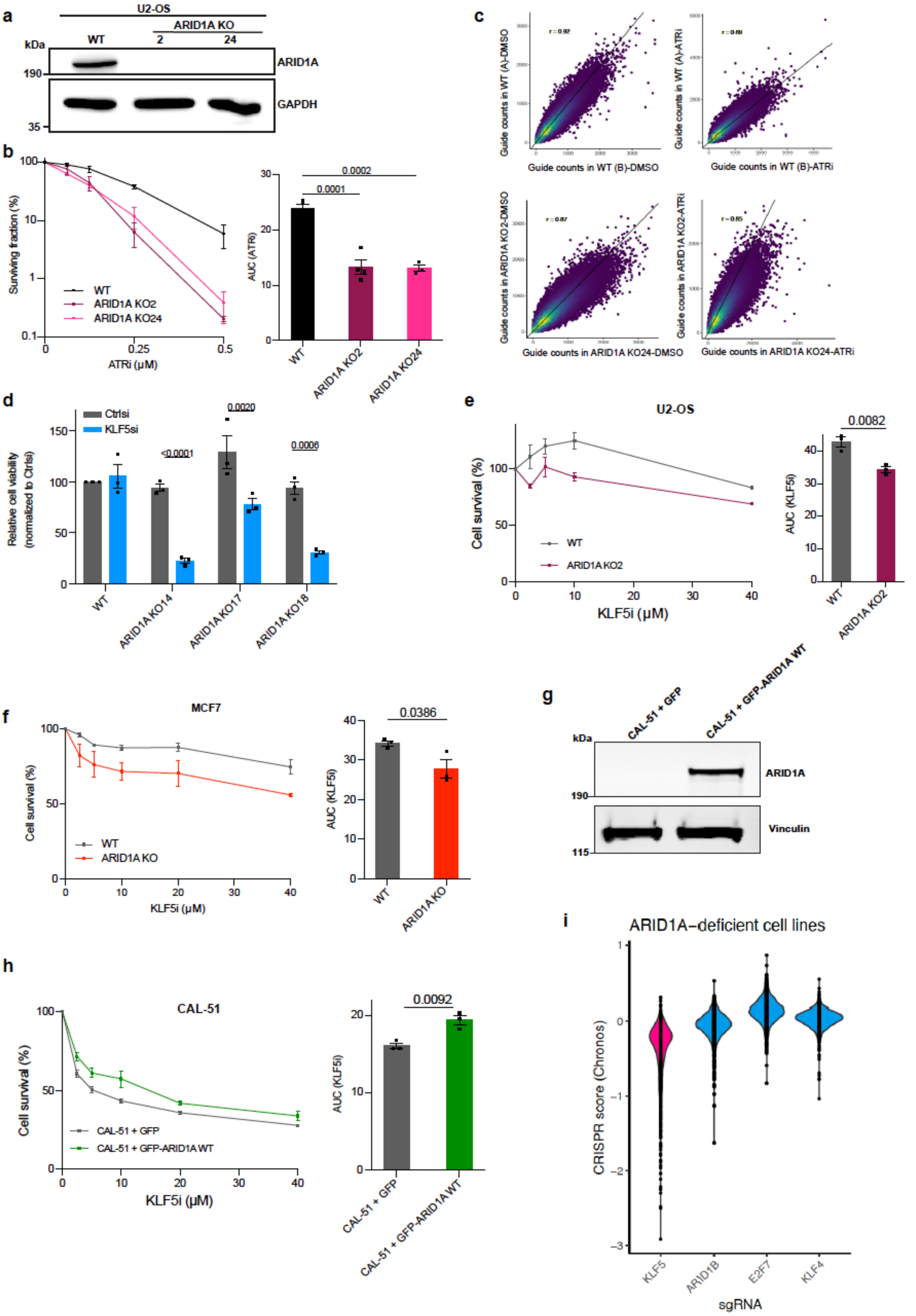

Supplementary Figure 1. Additional validation that loss of KLF5 sensitizes ARID1A-deficienct cells. a Generation of U2-OS *ARID1A* KO clones. Western blot analysis validating ARID1A protein levels in U2-OS cells following CRISPR-mediated gene knockout. GAPDH was used as a loading control. Representative of at least 3 independent experiments. **b** Clonogenic survivals of WT and *ARID1A* KO U2-OS treated with ATRi (AZD6738). Data represented as AUCs. Error bars represent means ± SEM, (biological n=4 for WT and ARID1A KO2, and n=3 for ARID1A KO24). Statistical analyses were performed by a one-way ANOVA test with multiple comparisons. **c** Correlation in read counts for WT cells treated with DMSO (upper left), WT cells treated with ATRi (upper right), *ARID1A* KO cells treated with DMSO (bottom left), and *ARID1A* KO cells treated with ATRi (bottom right). r, Spearman correlation coefficient. **d** alamarBlue cell viability assay of WT and three clones of *ARID1A* KO RPE-1 cells treated with CtrlsiRNA or KLF5siRNA. Error bars represent means with SEMs from 3 independent experiments. Statistical analyses were performed using two-way ANOVA test. **e, f** alamarBlue cell viability assay of WT and *ARID1A* KO U2-OS (e) and MCF-7 (f) cells treated with KLF5 inhibitor. Error bars represent means ± SEM, (biological n=3). Data represented as AUCs. Statistical analyses were performed using unpaired, two-sided *t*-test. **g** Immunoblot analysis of CAL-51 cells expressing GFP-only or GFP-ARID1A WT**.** Vinculin was used as a loading control. **h** alamarBlue cell viability assay of CAL-51 cells expressing either GFP-only or GFP-ARID1A WT treated with KLF5 inhibitor. Error bars represent means ± SEM, (biological n=3). Data represented as AUCs. Statistical analyses were performed using unpaired, two-tailed *t*-test. **i** Effect of different CRISPR knockouts in A*RID1A*-deficient cell lines on DepMap. Cell lines were classified as being ARID1A deficient if they had either: at least one ARID1A variant classed as "high" or "moderate" according to Variant Effect Predictor (VEP); or ARID1A mRNA expression lower than three median absolute deviations below the median ARID1A expression level. Points represent the CRISPR activity scores (Chronos) of the specified CRISPR knockouts for each ARID1A-deficient cell line. Source data are provided as a Source Data file.

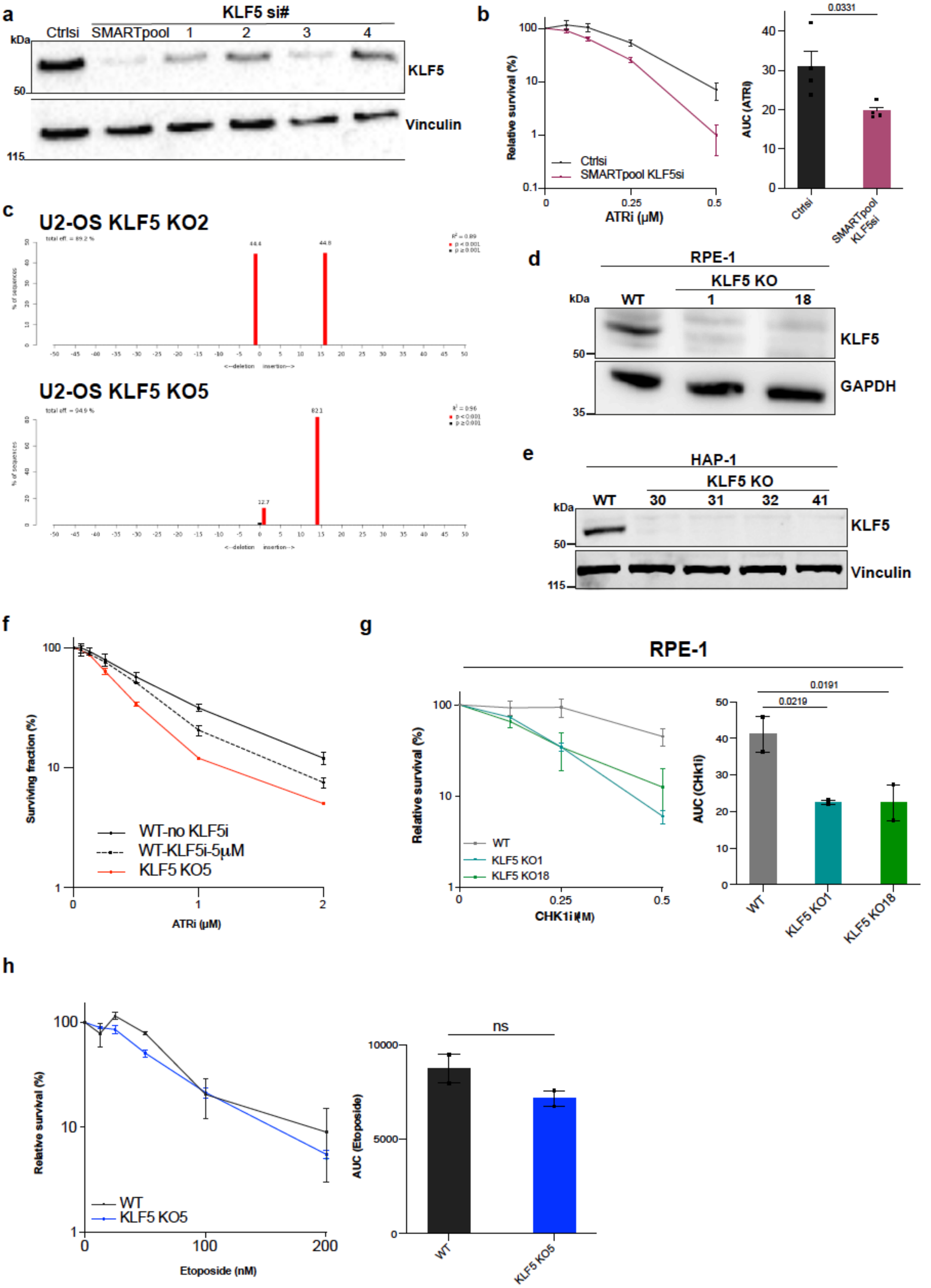

Supplementary Figure 2. Additional validation that KLF5 depletion sensitizes cells to replication stress. a Western blot analysis validating KLF5 protein levels in U2-OS cells following siRNA depletion. Vinculin was used as a loading control.

Representative of 3 independent experiments. **b** Clonogenic survivals of Ctrl and KLF5 siRNA-depleted U2-OS cells treated with ATRi (AZD6738). Data represented as AUCs. Error bars represent means ± SEM, (biological n=4), statistical analyses were performed using unpaired, two-sided *t*-test. **c** TIDE analysis of *KLF5* KO Cas9 editing in U2-OS cells. **d, e** Western blot validating *KLF5* KO in RPE-1 cells (d) and HAP-1 (e) cells. GAPDH/Vinculin were used as loading controls. **f** alamarBlue cell viability assay of U2-OS cells upon treatment with either ATRi only, or ATRi combined with KFL5i. *KLF5* KO cells (red) were used a positive control for sensitivity towards ATRi. Bars represent means ± SEM. **g** Clonogenic survivals of WT and *KLF5* KO RPE-1 cells treated with CHK1i. Data represented as AUCs. Data represented as AUCs. Error bars represent means ± SEM, (biological n=2, each with 3 technical replicates). Statistical analyses were performed using unpaired, two-sided *t*-test. **h** Clonogenic survivals of WT and *KLF5* KO U2-OS cells treated with etoposide. Data represented as AUCs. Error bars represent means ± SEM, (biological n=2, each with 3 technical replicates). Statistical analyses were performed using a one-way ANOVA test with multiple comparisons. Source data are provided as a Source Data file.

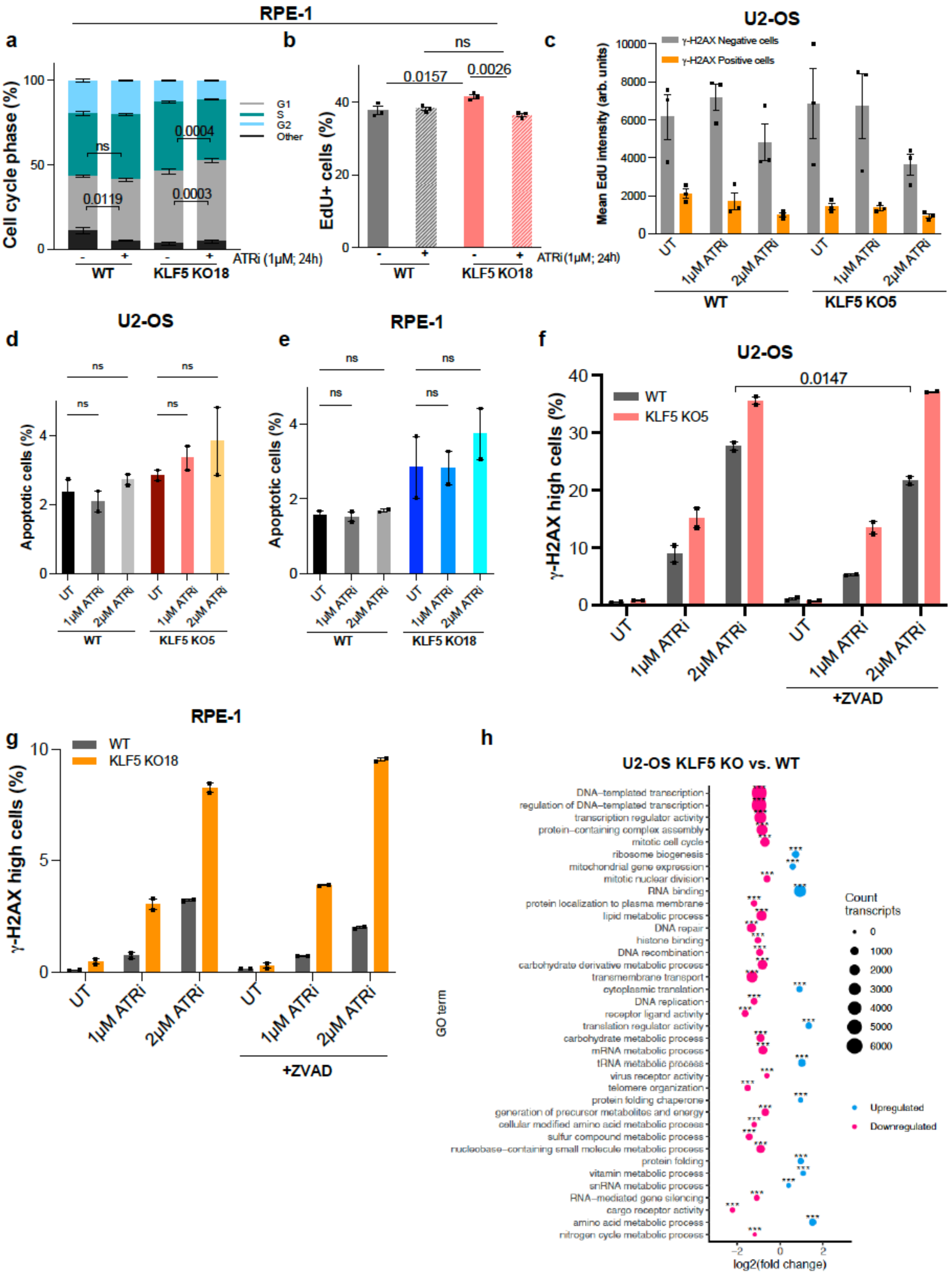

Supplementary Figure 3. Loss of KLF5 induces DNA damage upon ATRi. a Bar graphs showing percentages of cells in each cell cycle phase based on EdU and DAPI staining of WT and *KLF5* KO18 RPE-1 cells treated with DMSO or ATRi. Data are shown as means ± SEM from three independent biological experiments. Statistical

analyses were performed using two-way ANOVA test with multiple comparisons. **b** Bar graphs showing percentages of EdU positive WT and *KLF5* KO18 RPE-1 cells following ATRi treatment. Cells were labelled with 10uM EdU for 30 min prior to fixation. Data are shown as means ± SEM; biological *n* =3. Statistical analyses were performed using one-way ANOVA test. **c** Bar graphs showing mean EdU intensity of WT and KLF5 KO5 U2-OS S-phase γH2AX positive and S-phase γH2AX negative cells upon ATRi treatment. Data are shown as mean with SEM from three independent replicates. **d, e** Quantification of apoptotic cells in WT and KLF5 KO U2-OS (c) and RPE-1 (d) cells treated with the indicated concentrations of ATRi for 24h. Cells were stained with Annexin V and propidium iodide and analysed by flow cytometry. Percentages of apoptotic cells were determined by % Annexin V–positive cells and data are presented as mean with SEM from two independent experiments, each with three technical repeats. Statistical analyses were performed using one-way ANOVA test. ns= not significant. **f, g** Quantification of γH2AX high cells in WT and *KLF5* KO U2-OS (e) and RPE-1(f) cells following the indicated treatments (cells with either untreated, treated with 1μM ATRi only for 24h, treated with 10μM ZVAD only for 24h, or co-treated with 1μM ATRi and 10μM ZVAD for 24h). Data represented as means with SEM (biological n=2, each with three technical replicates). Statistical significance was assessed using two-way ANOVA followed by Šídák's multiple comparisons test, and adjusted *P* values are indicated in the figure, unless not significant. **h** Gene Ontology (GO) term analyses of genes dysregulated upon KLF5 depletion. Upregulated (red dots) and downregulated (blue dots) transcripts which had an adjusted p-value < 0.05 in *KLF5* KO cells were included. Source data are provided as a Source Data file.

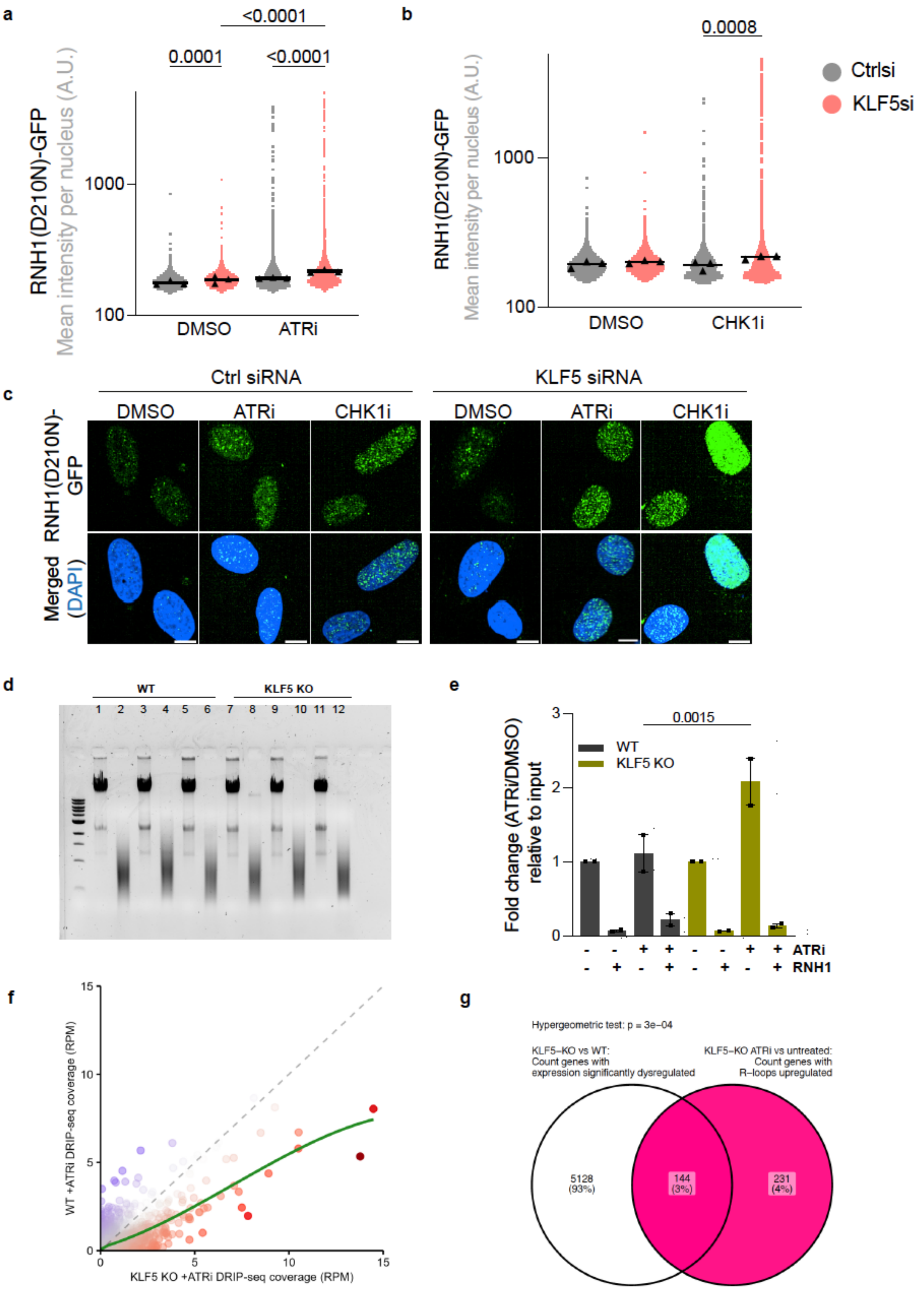

Supplementary Figure 4. KLF5 loss increases DNA-RNA hybrid formation upon ATR inhibition. a Formation of DNA-RNA hybrids was assessed by measuring the intensity of chromatin-bound catalytically inactive RNH1(D210N)-GFP in control or KLF5-depleted U2-OS cells either DMSO or ATRi-treated (1μM, 24h). **b** Same as in

a, except cells were treated with CHK1i. Mean intensities for each replicate are displayed as black triangles and were used for the overall mean calculations (biological n=3); statistical analyses were performed using one-way ANOVA test. **c** Representative images are of RNH1(D210N)-GFP following treatment with DMSO, 1uM ATRi for 24h, or 200nM CHK1i for 2h in control and KLF5-depleted cells. Scale bars = 10 µm. **d** Agarose gel validating DNA sonication fragmentation patterns for DRIP. Odd numbers show un-sonicated samples, and even numbers show sonicated samples. **e** Bar graph showing DRIP-qPCR analysis of the S9.6 changes in Actin B locus in WT and *KLF5* KO cells in untreated or ATRi treatment either untreated or pre-treated with RNH1. Two biological replicates of the DRIP were used for qPCR, each qPCR reaction was performed in three technical replicates. Statistical analyses were performed using one-way ANOVA test. **f** Correlation plot of DRIP signals in untreated conditions versus ATRi treatment between *KLF5* KO and WT U2-OS cells. **g** Venn diagram showing overlap of genes with dysregulated expression (either direction) and genes with increased R-loops. DRIP-seq peaks within gene body boundaries in human genome assembly GRCh38 according to NCBI RefSeq [1] were counted and compared using DESeq2 [2]. Concordance of lists of genes with differential R-loops and differential expression was tested for statistical significance using upper-tail hypergeometric tests. Source data are provided as a Source Data file.

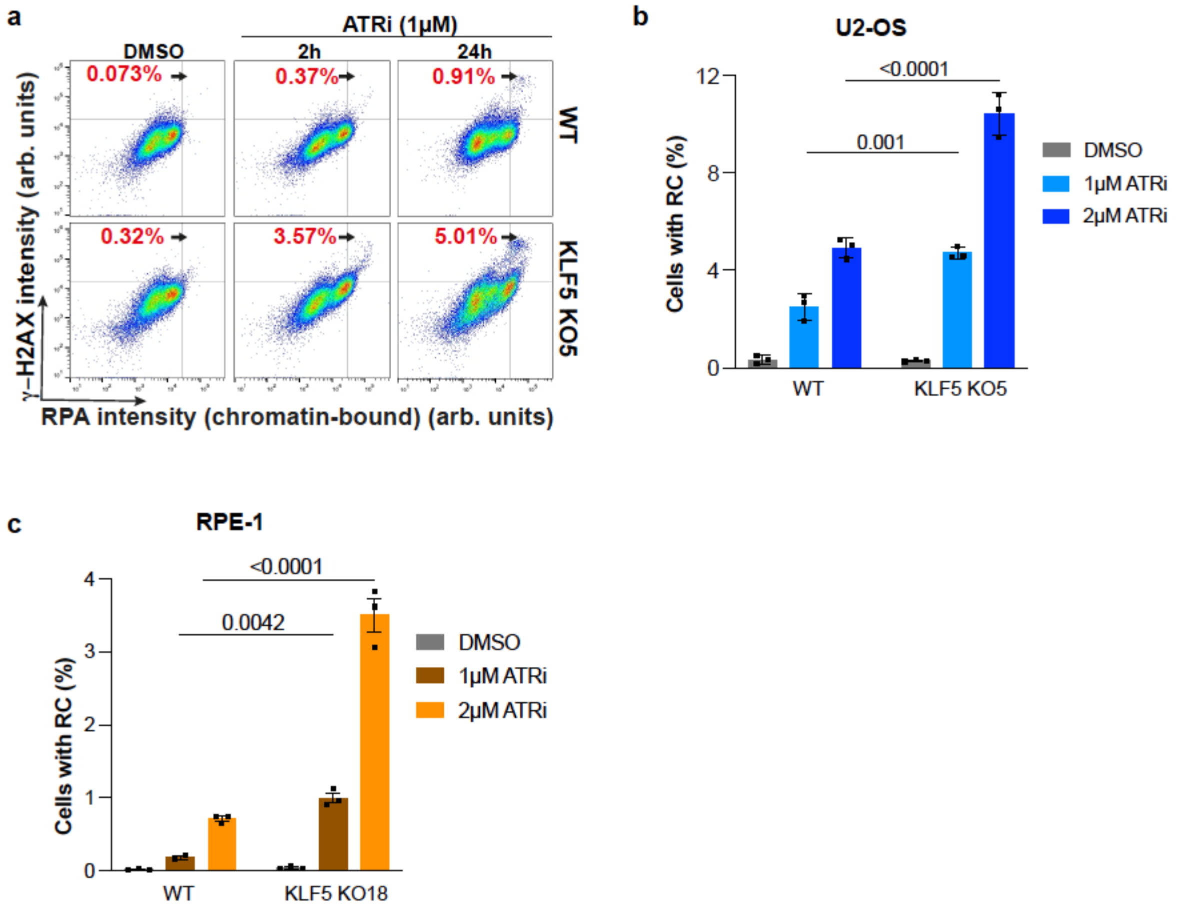

Supplementary Figure 5. KLF5 protects from replication stress. a FACS plots for percentages (in red) of RPA and γH2AX dual-positive cells (top right-hand quadrants) in WT and *KLF5* KO U2-OS cells treated with DMSO or 1 μM ATRi for the indicated times. **b, c** Quantification of cells with replication catastrophe upon ATRi at the indicated doses in WT and *KLF5* KO U2-OS (b) and RPE-1 (c). Data represented as means with SEM, biological n=3. Statistical analyses were performed using two-way ANOVA followed by Tukey's multiple comparisons test. Source data are provided as a Source Data file.

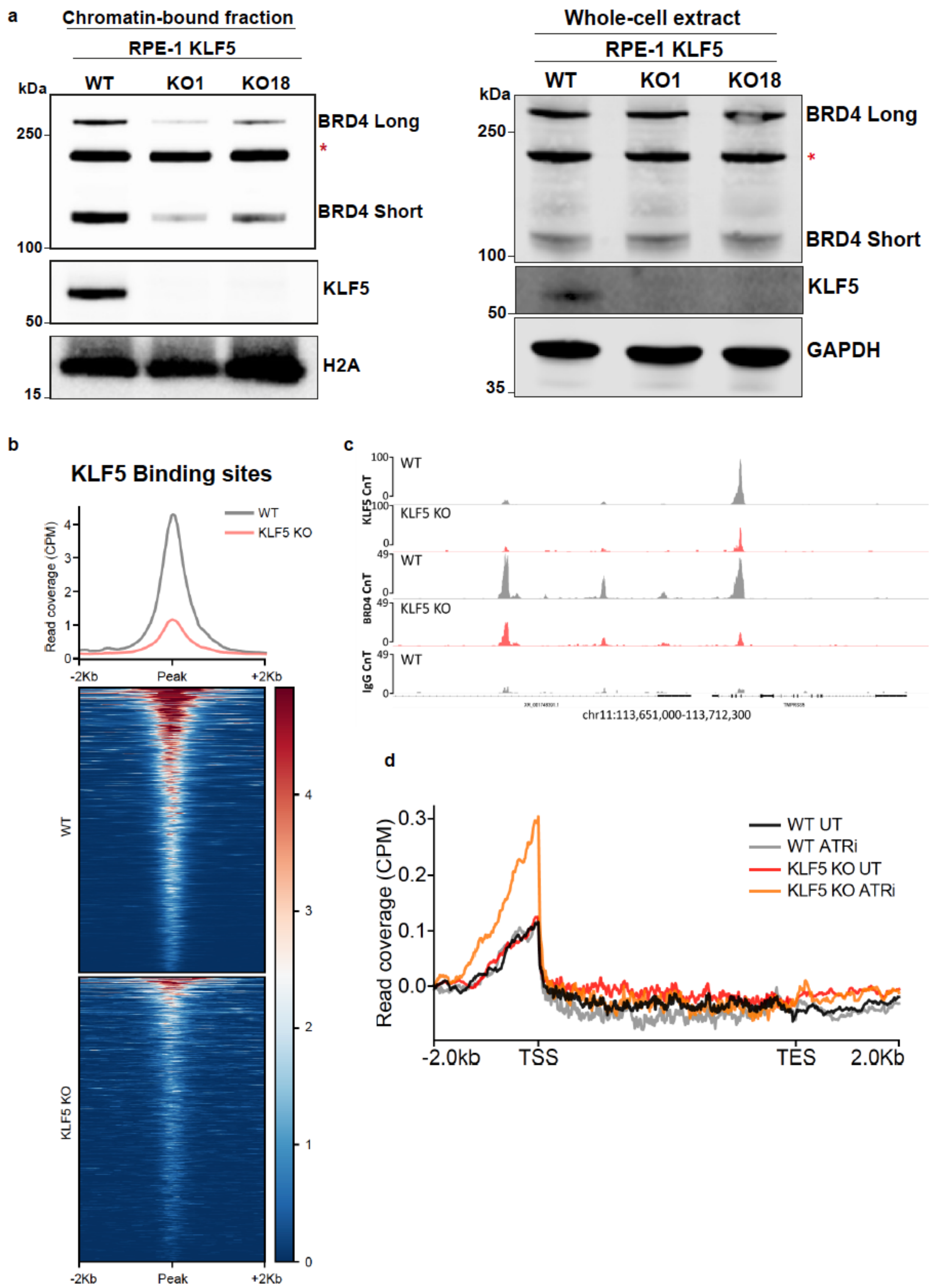

Supplementary Figure 6. KLF5 regulates the chromatin-recruitment of BRD4. a Left: western blot analysis of chromatin-bound BRD4 long and short isoforms in WT and *KLF5* KO RPE-1 cells. Histone H2A was used as marker for chromatin-bound fraction. Right: whole-cell extracts, the samples derive from the same experiment but

different gels, one gel for BRD4 and GAPDH, and another gel for KLF5.*- cross-reacting band. **b** Metagene (upper panel) and heat map (lower panel) demonstrating KLF5 binding sites in U2-OS cells. KLF5 binding sites were called based on WT relative to *KLF5* KO cells, (n=3). **c** Genome browser views of KLF5 CUT&Tag (CnT) (upper two panels), BRD4 CnT (middle two panels), and the negative control using IgG antibody (lower panel) in WT (grey peaks) and *KLF5* KO (red peaks) U2-OS cells. **d** Metagene plot of the distribution profile of S9.6 signals along all genes close to BRD4 binding sites and flanking regions (±2 kb) in WT and *KLF5* KO U2-OS cells treated with either DMSO or ATRi. Source data are provided as a Source Data file.

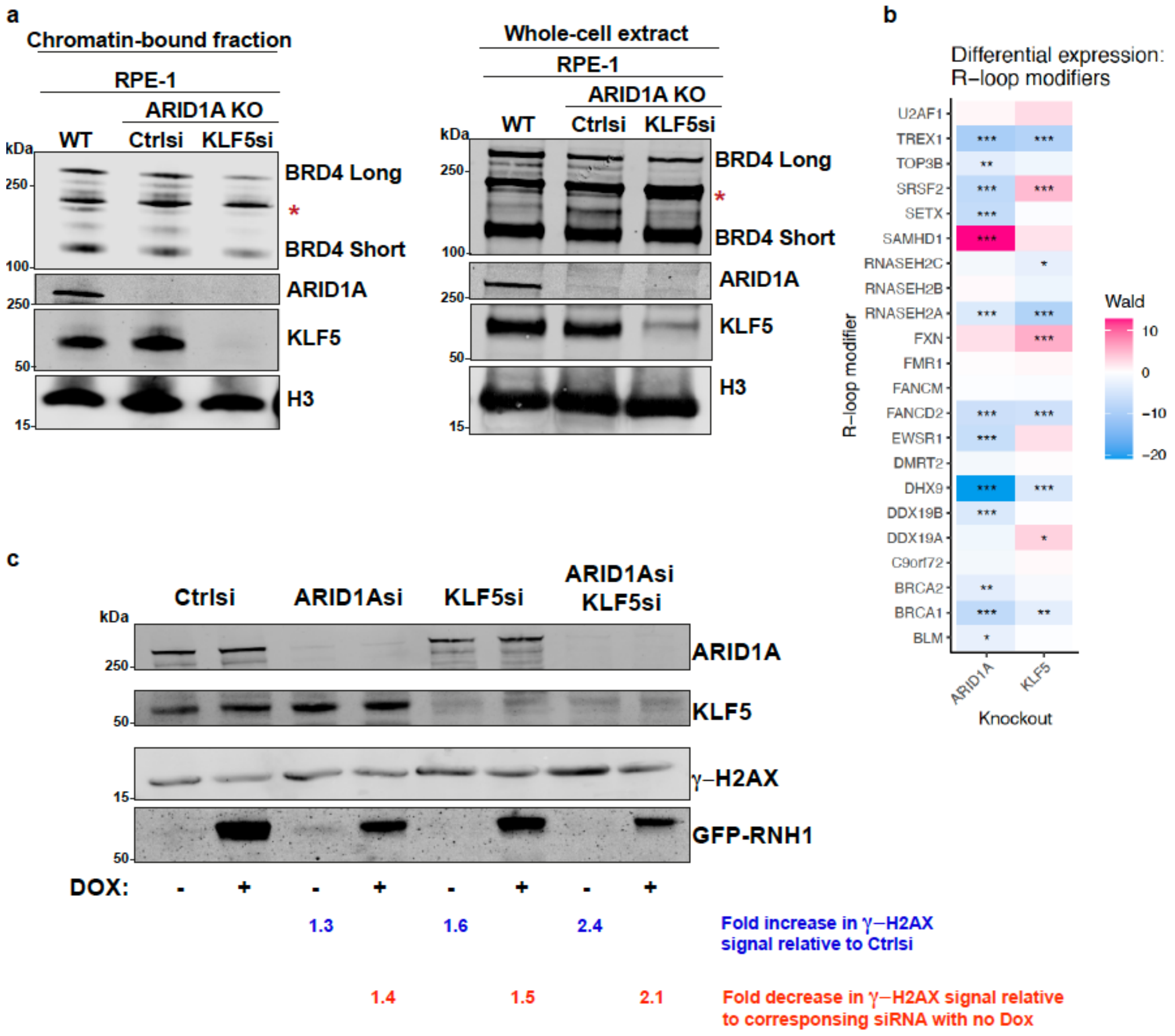

Supplementary Figure 7. KLF5 loss exacerbates R-loop-dependent DNA damage and is lethal for ARID1A-null cells. a Left: western blot analysis of chromatin-bound BRD4 long and short isoforms in WT and *ARID1A* KO RPE-1 cells transfected either with ctrl siRNA or siRNA targeting KLF5. Histone H3 was used as marker for chromatin-bound fraction. Right: whole-cell extracts. *- cross-reacting band. Representative of 4 independent experiments. **b** Differential expression of known R-loop modifier genes in *ARID1A* and *KLF5* knockout U2-OS cells. *, $p < 0.05$; **, $p < 0.01$; ***, $p < 0.001$. **c Top:** immunoblotting analysis of the DNA damage marker, γH2AX in control, KLF5 siRNA depleted, ARID1A siRNA depleted, or KLF5 and ARID1A double-depleted cells, in the absence or presence of DOX-induced RNaseH1 expression. Representative of 3 independent experiments **Bottom:** measurement of γH2AX signal intensity. Shown in blue is the fold increase in γH2AX signal in ARID1A siRNA depleted, KLF5 siRNA depleted, and ARID1A/KLF5 double-siRNA depleted cells. γH2AX intensity was normalized to control cells. Shown in red is the fold decrease in γH2AX signal upon DOX-induced expression of WT RNaseH1. γH2AX intensity was normalized to the corresponding cell line with no Dox. Source data are provided as a Source Data file.

Supplementary Tables:

Supplementary Table 1. List of antibodies used in this study.

Target	Supplier	Catalogue number	Application	Dilution	Species
ARID1A	Cell signalling	CST12354	Western blot	1:1000	rabbit
KLF5	abcam	ab137676	Western blot	1:1000	rabbit
GAPDH	Millipore	MAB374	Western blot	1:5000	mouse
Vinculin	abcam	ab219649	Western blot	1:1000	rabbit
ATM pS1981	Epitomics	2152-1	Western blot	1:2000	rabbit
ATM	abcam	ab32420	Western blot	1:2000	rabbit
CHK2 pT68	Cell signalling	CST2661	Western blot	1:1000	rabbit
CHK2	Cell signalling	CST2662	Western blot	1:1000	rabbit
RPA32 pS4/8	Cambridge Bioscience	A300-245A	Western blot	1:5000	rabbit
RPA32	Santa Cruz	sc-56770	Western blot	1:1000	mouse
γH2AX pS139	Cell signalling	CST2577	Western blot	1:2000	rabbit
H2AX	abcam	ab11175	Western blot	1:5000	rabbit
BRD4	abcam	ab128874	Western blot	1:1000	rabbit
Histone H3	abcam	ab1791	Western blot	1:5000	rabbit
H2A	abcam	Ab18255	Western blot	1:1000	rabbit
GFP	Santa Cruz	sc-9996	Western blot	1:1000	mouse
γH2AX pS139	Cell signalling	CST2577	FACS	1:200	rabbit
RPA32	abcam	ab2175	FACS	1:200	mouse
γH2AX pS139	Cell signalling	CST2577	IF	1:2500	rabbit
RPA32	abcam	ab2175	IF	1:300	mouse
PCNA	Santa Cruz	sc-56	PLA	1:2000	mouse
RNAPII-S2	Novus Biologicals	NB100-1805	PLA	1:2000	rabbit
S9.6	millipore	Mabe1095	DRIP	10 μg/sample	mouse
KLF5	abcam	ab137676	CnT	1:50	rabbit
BRD4	Cambridge Bioscience	A700-004	CnT	1:50	rabbit
IgG	CST	CSt2729S	CnT	1:50	rabbit

Supplementary Table 2. List of sgRNAs and primers used in this study.

Target	sgRNA sequence (5'-3')	FW primer (5'-3')	RV primer (5'-3')
ARID1A	CAATAGATGACCTCCCCATG	GTCTGACTACTGTTGCACTATAGTG	CTGGATCTCTTTGTGTGTGATACTG
KLF5	CTCATTTAAAAGCTCACCTG	GCCTTTAATAAGGTAATGGGGGAG	GCTTGTAACCCATTTCTGACCAC
Primers used in DRIP-qPCR			
ActinB		CAACTGGGACGACATGGAGAAA	GAGTCCTACGGAAAACGGCAGA

Supplementary Table 3. List of siRNAs used in this study.

siRNA	Target RNA sequence	Target gene
KLF5 (SMARTpool)	CCAGAGACCGUGCGUAACA (si1)	KLF5
	GAACUGGCCUCUACAAAUC (si2)	
	CAACCUGUCAGAUACAAUA (si3)	
	GCAGCAAUGGACACUCUUA (si4)	
ARID1A (SMARTpool)	GAAUAGGGCCUGAGGGAAA (si5)	ARID1A
	AGAUGUGGGUGGACCGUUA (si6)	
	GCAACGACAUGAUUCCUAU (si7)	
	GGACCUCUAUCGCCUCUAU (si8)	
BRD4 (SMARTpool)	AAACCGAGAUCAUGAUAGU (si6)	BRD4
	CUACACGACUACUGUGACA (si7)	

	AAACACAACUCAAGCAUCG (si8)	
	CAGCGAAGACUCCGAAACA (si9)	

Supplementary Methods:

AlamarBlue cell survival assays

AlamarBlue (resazurin) cell viability assays were performed as previously described [3]. 2000 U2-OS cells were plated in 96-well plates in triplicate. 16h later, cells were treated with the indicated drugs for five days. At the end of the treatment, 0.1× volume of alamarBlue was added to each well, incubated for 3-4h at 37°C in a tissue culture incubator, and absorbance was read at an excitation wavelength of 530/560 nm and an emission wavelength of 590 nm.

Annexin-V apoptosis assay

Apoptosis was assessed by staining cells with Annexin V and propidium Iodide solution (Invitrogen, V13245) followed by flow cytometry analysis. WT and KLF5 KO U2-OS and RPE-1 cells were treated with ATRi at the indicated concentrations for 24h prior to staining. Samples were acquired using A5 FACS Symphony (BD Biosciences) and analysed in FlowJo v.10.8.1.

RNA-seq

WT, *KLF5* KO, and *ARID1A* KO U2-OS cells were used in two biological replicates for RNA sequencing (RNA-seq). 16h following seeding in 10 cm^2 dishes, cells were trypsinized, washed with PBSx1, and total RNA was extracted using the RNeasy kit with on column DNase digestion according to the manufacturer's protocol (Qiagen). RNA integrity was confirmed with Agilent Tapestation RNA screen Tape. Ribosomal RNA was depleted from mRNA, and following library preparation, sequencing was performed using single-ended reads on an Illumina NovaSeq6000. Transcript abundances were quantified using kallisto[4] software by pseudoalignment with Ensembl 108 (Martin et al, 2023). DESeq2 [2] software was used to identify differentially expressed genes in *KLF5* KO, or *ARID1A* KO compared to WT. Functional analysis by gene set enrichment analysis was performed using piano [5] software with Gene Ontology (GO) terms as gene sets. GO terms were combined into a custom ontology (Table S5) for display purposes.

DRIP-qPCR

Enriched chromatin from DRIP was analysed by qPCR. qPCR was done in a final volume of 20μl/reaction by using 2μl of 1:10 diluted DRIP chromatin, 10μl of 2x Fast SYBR Green Master mix (Thermo Fisher Scientific) and 300nM forward and reverse primers (qPCR primers are listed in Supplementary Table 2). For each biological replicate, qPCR was done in technical triplicates. The QuantStudio 5 Real-Time PCR

640 System (Thermo Fisher Scientific) was used to obtain raw Cq values, which were then used for further analysis.

Supplementary References:

1 O'Leary, N. A. *et al.* Reference sequence (RefSeq) database at NCBI: current status, taxonomic expansion, and functional annotation. *Nucleic Acids Res* **44**, D733-745, doi:10.1093/nar/gkv1189 (2016).

2 Love, M. I., Huber, W. & Anders, S. Moderated estimation of fold change and dispersion for RNA-seq data with DESeq2. *Genome Biol* **15**, 550, doi:10.1186/s13059-014-0550-8 (2014).

3 Kumar, P., Nagarajan, A. & Uchil, P. D. Analysis of Cell Viability by the alamarBlue Assay. *Cold Spring Harb Protoc* **2018**, doi:10.1101/pdb.prot095489 (2018).

4 Bray, N. L. Fast RNA-seq quantification. *Nature Methods* **13**, 470-470, doi:10.1038/nmeth.3879 (2016).

5 Varemo, L., Nielsen, J. & Nookaew, I. Enriching the gene set analysis of genome-wide data by incorporating directionality of gene expression and combining statistical hypotheses and methods. *Nucleic Acids Res* **41**, 4378-4391, doi:10.1093/nar/gkt111 (2013).
