# Supplementary material for: KLF5 loss sensitizes cells to ATR inhibition and is synthetic lethal with ARID1A deficiency
Source: Nat Commun. 2025 Jan 8;16:480. doi: 10.1038/s41467-024-55637-5 (PMC11711288; doi:10.1038/s41467-024-55637-5)
Supplement: Supplementary file 2 — Description of Additional Supplementary Files [file 41467_2024_55637_MOESM2_ESM.pdf]

Description of Additional Supplementary Files

File Name: Supplementary Data 1

Description: An Excel file containing NormZ score outputs from DrugZ analyses of CRISPR screens, related to Figure 1.

File Name: Supplementary Data 2

Description: An Excel file containing differential expression and gene set enrichment analysis in *KLF5* KO clone 5, and *ARID1A* KO clone 2 versus wildtype, related to Supplementary Figure 3 and 7.
