# Supplementary material for: KLF5 loss sensitizes cells to ATR inhibition and is synthetic lethal with ARID1A deficiency
Source: Nat Commun. 2025 Jan 8;16:480. doi: 10.1038/s41467-024-55637-5 (PMC11711288; doi:10.1038/s41467-024-55637-5)
Supplement: Supplementary file 5 — Reporting Summary [file 41467_2024_55637_MOESM5_ESM.pdf]

nature portfolio

Corresponding author(s): Samah W. Awwad
Stephen P. Jackson

Last updated by author(s): Nov 28, 2024

Reporting Summary

Nature Portfolio wishes to improve the reproducibility of the work that we publish. This form provides structure for consistency and transparency in reporting. For further information on Nature Portfolio policies, see our Editorial Policies and the Editorial Policy Checklist.

Statistics

For all statistical analyses, confirm that the following items are present in the figure legend, table legend, main text, or Methods section.

n/a	Confirmed	
☐	☒	The exact sample size (n) for each experimental group/condition, given as a discrete number and unit of measurement
☒	☐	A statement on whether measurements were taken from distinct samples or whether the same sample was measured repeatedly
☐	☒	The statistical test(s) used AND whether they are one- or two-sided<br>*Only common tests should be described solely by name; describe more complex techniques in the Methods section.*
☒	☐	A description of all covariates tested
☒	☐	A description of any assumptions or corrections, such as tests of normality and adjustment for multiple comparisons
☐	☒	A full description of the statistical parameters including central tendency (e.g. means) or other basic estimates (e.g. regression coefficient) AND variation (e.g. standard deviation) or associated estimates of uncertainty (e.g. confidence intervals)
☐	☒	For null hypothesis testing, the test statistic (e.g. F, t, r) with confidence intervals, effect sizes, degrees of freedom and P value noted<br>*Give P values as exact values whenever suitable.*
☒	☐	For Bayesian analysis, information on the choice of priors and Markov chain Monte Carlo settings
☒	☐	For hierarchical and complex designs, identification of the appropriate level for tests and full reporting of outcomes
☒	☐	Estimates of effect sizes (e.g. Cohen's d, Pearson's r), indicating how they were calculated

Our web collection on statistics for biologists contains articles on many of the points above.

Software and code

Policy information about availability of computer code

Data collection	Western blotting images were captured using ChemiDoc MP Imaging Sysytem (Bio-Rad). Flow cytometry analysis was performed using Fortessa (BD biosciences). High-throughput image data were acquired on Opera Phenix microscope using Harmony High-Content Imaging and Analysis software (Perkin Elmer).
Data analysis	Genome wide CRISPR screens were analysed using DrugZ. Transcript abundances were quantified using kallisto software by pseudo alignment with Ensembl 108. DESeq2 software was used to identify differentially expressed genes in KLF5 KO compared to WT. Functional analysis by gene set enrichment analysis was performed using piano software with Gene Ontology (GO) terms as gene sets. GO terms were combined into a custom ontology (Table S5) for display purposes. Flow cytometry data was analyzed using FlowJo v.10.8.1. Graphs were generated using Graphpad Prism version 10. Figures were prepared using Adobe Illustrator. The following software was used: fastp v0.22.0, bowtie2 v2.2.5, MACS2 v2.2.6, deepTools v3.5.1, BEDTools v2.30.0

For manuscripts utilizing custom algorithms or software that are central to the research but not yet described in published literature, software must be made available to editors and reviewers. We strongly encourage code deposition in a community repository (e.g. GitHub). See the Nature Portfolio guidelines for submitting code & software for further information.

Data

Policy information about availability of data

All manuscripts must include a data availability statement. This statement should provide the following information, where applicable:

- Accession codes, unique identifiers, or web links for publicly available datasets
- A description of any restrictions on data availability
- For clinical datasets or third party data, please ensure that the statement adheres to our policy

Sequencing reads for the CRISPR screen have been deposited in ENA under accession number PRJEB75512RNA sequencing data generated in this study have been deposited in European Nucleotide Archive (ENA) under accession number PRJEB75523. DRIP-seq raw data generated in this study have been deposited with Array Express under the accession code E-MTAB-14062. Cut and Tag raw data generated in this study have been deposited with Array Express under the accession code E-MTAB-14079. All relevant data are available from the corresponding authors.

Research involving human participants, their data, or biological material

Policy information about studies with human participants or human data. See also policy information about sex, gender (identity/presentation), and sexual orientation and race, ethnicity and racism.

Reporting on sex and gender	NA
Reporting on race, ethnicity, or other socially relevant groupings	NA
Population characteristics	NA
Recruitment	NA
Ethics oversight	NA

Note that full information on the approval of the study protocol must also be provided in the manuscript.

Field-specific reporting

Please select the one below that is the best fit for your research. If you are not sure, read the appropriate sections before making your selection.

☒ Life sciences ☐ Behavioural & social sciences ☐ Ecological, evolutionary & environmental sciences

For a reference copy of the document with all sections, see nature.com/documents/nr-reporting-summary-flat.pdf

Life sciences study design

All studies must disclose on these points even when the disclosure is negative.

Sample size	CRISPR screen was performed using two different ARID1A KO clones, with two technical replicates. Sample sizes for in vitro experiments were not determined by statistical power analysis but chosen according to standard practices in the field. Immunoblots were repeated at least three times with one representative experiment presented, as indicated in the figure legends.
Data exclusions	No data were excluded.
Replication	Sample size and number of replicates in each experiment is mentioned in the figure legend and or/methods section. At least two independent clones of genetically modified (using CRISPR technology) cell lines were used for each assay. in addition to the biological replicates, we show that loss of KLF5 sensitizes cells to ATR inhibition in three different cells lines, U2-OS, RPE-1 and HAP1-1 cells, and validates the synthetic lethality between KLF5 and ARID1A in multiple cells lines (U2-OS, RPE-1, MCF-7 and CAL-51) using multiple assays.
Randomization	For microscopy-based analysis, a fixed number of images were obtained randomly from each sample and analyzed together with control samples using the same analysis pipeline.
Blinding	No blinding was used during sample collection and processing. However, unless indicated, image acquisition and data analyses were performed in an unbiased way using high-throughput high-content Opera Phenix microscope and Harmony software.

Reporting for specific materials, systems and methods

We require information from authors about some types of materials, experimental systems and methods used in many studies. Here, indicate whether each material, system or method listed is relevant to your study. If you are not sure if a list item applies to your research, read the appropriate section before selecting a response.

Materials & experimental systems

n/a	Involved in the study	
	x	Antibodies
	x	Eukaryotic cell lines
x		Palaeontology and archaeology
x		Animals and other organisms
x		Clinical data
x		Dual use research of concern
x		Plants

Methods

n/a	Involved in the study	
	x	ChIP-seq
	x	Flow cytometry
x		MRI-based neuroimaging

Antibodies

Antibodies used

ARID1A Cell signaling CST12354
KLF5 abcam ab137676
GAPDH Millipore MAB374
Vinculin abcam ab219649
BRD4 abcam ab128874
Histone H3 abcam ab1791
gH2AX pS139 Cell signalling CST2577
RPA32 abcam ab2175
gH2AX pS139 Cell signalling CST2577
RPA32 abcam ab2175
PCNA Santa Cruz sc56
RNAPII-S2 Novus Biologicals NB100-1805
S9.6 millipore Mabe1095
KLF5 abcam ab137676
BRD4 Cambridge Bioscience A700-004
IgG CST CSt2729S
ATM pS1981 Epitomics 2152-1
ATM abcam ab32420
CHK2 pT68 Cell signalling CST2661
CHK2 Cell signalling CTS2662
RPA32 pS4/8 Cambridge Bioscience A300-245A
RPA32 Santa cruz sc-56770
H2AX abcam ab11175

Validation

Commercially available antibodies were validated by the supplier and by us using appropriate controls. Please see more details in the manufacturers' websites for further details. Each experiment had appropriate controls to validate the antibodies, i.e. siRNA or CRISPR-mediated KO. antibodies against damage-specific phosphorylations (pATM, gH2AX, pCHK2, pRPA32) were validated by the induction of the expected band/intensityfollowing replication stress induction by ATRi/CHK1i.

Eukaryotic cell lines

Policy information about cell lines and Sex and Gender in Research

Cell line source(s)

U2-OS Cas9 U2-OS WT were originally obtained from ATCC, and then used to generate U2-OS stablly expressing Cas9 (#71489)
U2-OS ARID1A KO This study
U2-OS KLF5 KO This study
RPE-1 Cas9 RPE-1 originally obtained from J.Pines [], and then used to generate RPE-1stablly expressing Cas9 (#71489)
RPE-1 KLF5 KO This study
RPE-1 ARID1A KO this study
HAP-1 KLF5 KO this study
LentiX 293T Takara Bio
U2-OS T-Rex GFP-RNase H1(D210N) kind gift from Pavel Janscak
GFP-RNase H1 (RNH1-GFP) kind gift from Pavel Janscak
MCF-7 kind gift from Jason Carroll
CAL-51 SPJ lab

Authentication

All KO cell lines used and/or generated in this study were validated by western blot, PCR using specific primers and sequencing.

Mycoplasma contamination

All cell lines used in this study were routinely tested for mycoplasma contamination.

Commonly misidentified lines (See ICLAC register)

No commonly misidentified cell lines were used

Plants

Seed stocks

NA

Novel plant genotypes

NA

Authentication

NA

ChIP-seq

Data deposition

☒ Confirm that both raw and final processed data have been deposited in a public database such as GEO.

☒ Confirm that you have deposited or provided access to graph files (e.g. BED files) for the called peaks.

Data access links
May remain private before publication.

Data accessible via ArrayExpress with the accession code E-MTAB-14062

Files in database submission

U2OS_KLF5KO5_ATRi_1.bw
U2OS_KLF5KO5_ATRi_2.bw
U2OS_KLF5KO5_ATRi_3.bw
U2OS_KLF5KO5_UT_1.bw
U2OS_KLF5KO5_UT_2.bw
U2OS_KLF5KO5_UT_3.bw
U2OS_WT_ATRi_1.bw
U2OS_WT_ATRi_2.bw
U2OS_WT_ATRi_3.bw
U2OS_WT_UT_1.bw
U2OS_WT_UT_2.bw
U2OS_WT_UT_3.bw
U2OS_KLF5KO5_ATRi_1_r1.fq.gz
U2OS_KLF5KO5_ATRi_1_r2.fq.gz
U2OS_KLF5KO5_ATRi_2_r1.fq.gz
U2OS_KLF5KO5_ATRi_2_r2.fq.gz
U2OS_KLF5KO5_ATRi_3_r1.fq.gz
U2OS_KLF5KO5_ATRi_3_r2.fq.gz
U2OS_KLF5KO5_UT_1_r1.fq.gz
U2OS_KLF5KO5_UT_1_r2.fq.gz
U2OS_KLF5KO5_UT_2_r1.fq.gz
U2OS_KLF5KO5_UT_2_r2.fq.gz
U2OS_KLF5KO5_UT_3_r1.fq.gz
U2OS_KLF5KO5_UT_3_r2.fq.gz
U2OS_WT_ATRi_1_r1.fq.gz
U2OS_WT_ATRi_1_r2.fq.gz
U2OS_WT_ATRi_2_r1.fq.gz
U2OS_WT_ATRi_2_r2.fq.gz
U2OS_WT_ATRi_3_r1.fq.gz
U2OS_WT_ATRi_3_r2.fq.gz
U2OS_WT_UT_1_r1.fq.gz
U2OS_WT_UT_1_r2.fq.gz
U2OS_WT_UT_2_r1.fq.gz
U2OS_WT_UT_2_r2.fq.gz
U2OS_WT_UT_3_r1.fq.gz
U2OS_WT_UT_3_r2.fq.gz

Genome browser session (e.g. UCSC)

All data is available in graphable formats at the accession link for use directly in genome browsers.

Methodology

Replicates	In this study we performed DRIP-seq in 3 independent biological replicates
Sequencing depth	Samples were sequenced to an average depth of ~112 million reads with >99% passing quality filters with fastp and ~105 million reads successfully aligning to the hg38 human genome. All reads were paired-end 50 cycles.
Antibodies	S9.6 millipore Mabe1095 DRIP 10 ug/sample
Peak calling parameters	MACS2 callpeak was used with the following parameters: "-g 2.86e9 -f BAMPE --keep-dup all --broad -B" and comparisons were made between untreated and ATRi treated conditions
Data quality	Peaks were filtered for an enrichment score of >=10
Software	The following software was used: fastp v0.22.0, bowtie2 v2.2.5, MACS2 v2.2.6, deepTools v3.5.1, BEDTools v2.30.0

Flow Cytometry

Plots

Confirm that:

- [x] The axis labels state the marker and fluorochrome used (e.g. CD4-FITC).
- [x] The axis scales are clearly visible. Include numbers along axes only for bottom left plot of group (a 'group' is an analysis of identical markers).
- [x] All plots are contour plots with outliers or pseudocolor plots.
- [x] A numerical value for number of cells or percentage (with statistics) is provided.

Methodology

Sample preparation	U2-OS or RPE-1 cells were pre-extracted with PBS-T (PBS supplemented with 0.2% Triton X-100) for 10 min on ice, followed by 15 min fixation permeabilization with BD Cytofix/Cytoperm (BD bioscience) at room temperature. Antibody staining was performed in BD perm/wash buffer (BD biosciences).
Instrument	Fortessa (BD biosciences) and A5 FACS Symphony (BD biosciences)
Software	FlowJo v.10.8.1.
Cell population abundance	Cell sorting was not performed in this study.
Gating strategy	FSC/SSC gates define single cell population.<br>For each condition, 30,000 single cells were recorded for downstream analysis.<br>To determine gH2AX positive, and RPA positive populations, gates were defined based on the untreated cell population. in the control cells.<br>For cell cycle analysis, 50,000 single cells were recorded.

- [x] Tick this box to confirm that a figure exemplifying the gating strategy is provided in the Supplementary Information.
